# A prognostic model for systemic lupus erythematosus-associated pulmonary arterial hypertension: CSTAR-PAH cohort study

**DOI:** 10.1186/s12931-023-02522-2

**Published:** 2023-09-09

**Authors:** Jingge Qu, Mengtao Li, Xiao Zhang, Miaojia Zhang, Xiaoxia Zuo, Ping Zhu, Shuang Ye, Wei Zhang, Yi Zheng, Wufang Qi, Yang Li, Zhuoli Zhang, Feng Ding, Jieruo Gu, Yi Liu, Junyan Qian, Can Huang, Jiuliang Zhao, Qian Wang, Yongtai Liu, Zhuang Tian, Yanhong Wang, Wei Wei, Xiaofeng Zeng

**Affiliations:** 1 Department of Rheumatology, Peking Union Medical College Hospital (PUMCH), Peking Union Medical College & Chinese Academy of Medical Sciences, National Clinical Research Center for Dermatologic and Immunologic Diseases (NCRC-DID), Ministry of Science & Technology, Key Laboratory of Rheumatology and Clinical Immunology, Ministry of Education, State Key Laboratory of Complex Severe and Rare Diseases, Ministry of Science and Technology, No. 1 Shuaifuyuan, Wangfujing Ave, Beijing, 100730 China; 2 Department of Respiratory and Critical Care Medicine, Peking University Third Hospital, Beijing, China; 3 Department of Rheumatology, Guangdong General Hospital, Guangzhou, China; 4 Department of Rheumatology, The First Affiliated Hospital of Nanjing Medical University, Nanjing, China; 5 Department of Rheumatology, Xiangya Hospital, Central South University, Changsha, China; 6 Department of Clinical Immunology, PLA Specialized Research Institute of Rheumatology and Immunology, Xijing Hospital, Fourth Military Medical University, Xi’an, China; 7 Department of Rheumatology, School of Medicine, Shanghai Jiao Tong University, Ren Ji Hospital South Campus, Shanghai, China; 8 Department of Rheumatology, School of Medicine, Ren Ji Hospital, Shanghai Jiao Tong University, Shanghai, China; 9 Department of Rheumatology, Beijing Chao-Yang Hospital, Capital Medical University, Beijing, China; 10 Department of Rheumatology, The First Central Hospital, Tianjin, China; 11 Department of Rheumatology, The Second Affiliated Hospital of Harbin Medical University, Harbin, China; 12 Department of Rheumatology and Clinical Immunology, Peking University First Hospital, Beijing, China; 13 Department of Rheumatology, Qilu Hospital of Shandong University, Jinan, China; 14 Department of Rheumatology, The Third Affiliated Hospital of Sun Yat-Sen University, Guangzhou, China; 15 Department of Rheumatology and Immunology, West China Hospital, Sichuan University, Chengdu, China; 16 Department of Cardiology, Peking Union Medical College Hospital, Peking Union Medical College & Chinese Academy of Medical Sciences, State Key Laboratory of Complex Severe and Rare Diseases, Ministry of Science & Technology, Beijing, China; 17 Department of Epidemiology and Bio-Statistics, Institute of Basic Medical Sciences, China Academy of Medical Sciences and Peking Union Medical College, Beijing, China; 18 Department of Rheumatology, Tianjin Medical University General Hospital, No. 154 Anshan Street, Tianjin, 300052 China

**Keywords:** Pulmonary arterial hypertension, Prognosis, Risk stratification, Systemic lupus erythematosus

## Abstract

**Background:**

Pulmonary arterial hypertension is a major cause of death in systemic lupus erythematosus, but there are no tools specialized for predicting survival in systemic lupus erythematosus-associated pulmonary arterial hypertension.

**Research question:**

To develop a practical model for predicting long-term prognosis in patients with systemic lupus erythematosus-associated pulmonary arterial hypertension.

**Methods:**

A prognostic model was developed from a multicenter, longitudinal national cohort of consecutively evaluated patients with systemic lupus erythematosus-associated pulmonary arterial hypertension. The study was conducted between November 2006 and February 2020. All-cause death was defined as the endpoint. Cox regression and least absolute shrinkage and selection operators were used to fit the model. Internal validation of the model was assessed by discrimination and calibration using bootstrapping.

**Results:**

Of 310 patients included in the study, 81 (26.1%) died within a median follow-up of 5.94 years (interquartile range 4.67–7.46). The final prognostic model included eight variables: modified World Health Organization functional class, 6-min walking distance, pulmonary vascular resistance, estimated glomerular filtration rate, thrombocytopenia, mild interstitial lung disease, N-terminal pro-brain natriuretic peptide/brain natriuretic peptide level, and direct bilirubin level. A 5-year death probability predictive algorithm was established and validated using the C-index (0.77) and a satisfactory calibration curve. Risk stratification was performed based on the predicted probability to improve clinical decision-making.

**Conclusions:**

This new risk stratification model for systemic lupus erythematosus-associated pulmonary arterial hypertension may provide individualized prognostic probability using readily obtained clinical risk factors. External validation is required to demonstrate the accuracy of this model's predictions in diverse patient populations.

**Supplementary Information:**

The online version contains supplementary material available at 10.1186/s12931-023-02522-2.

## Introduction

Pulmonary arterial hypertension (PAH) is a fatal condition and a leading cause of death among patients with systemic lupus erythematosus (SLE) [1]. The prevalence of PAH in patients with SLE is estimated to be < 5% [2]. However, with the high prevalence of SLE in Asian countries, SLE-associated PAH (SLE-PAH) accounts for a large proportion of connective tissue disease-associated PAH and group 1 PAH cases [3, 4]. Therefore, a reasonable assessment of prognosis and timely intervention in patients with SLE-PAH are quite necessary.

Currently, the approach to assessing patients with SLE-PAH relies on the recommendation for group 1 PAH, such as the 2015 and 2022 European Society of Cardiology (ESC) / European Respiratory Society (ERS) guidelines [5, 6], the risk scores derived from the ESC/ERS guideline risk table [7–9] and the Registry to Evaluate Early and Long-Term PAH Disease Management (REVEAL) prognostic tools [10]. However, SLE-PAH is rarely considered by these prognostic tools because of the limited number of cases. Even though the mode of death in SLE-PAH is predominately due to right ventricular failure. Multiorgan involvement of lupus is likely to worsen right ventricular failure due to PAH [11]. This needs to be taken into account when assessing the prognosis of SLE-PAH. Given this situation, prognosis prediction tools specific for SLE-PAH are needed in clinical practice.

The Chinese SLE Treatment and Research Group (CSTAR) was established and funded by the Chinese Ministry of Science and Technology in 2009 and was further extended with the formation of the Chinese Rheumatism Data Center, which is directed by the National Health and Family Planning Commission of China [12, 13]. The CSTAR-PAH cohort is a multicenter registry consisting of 14 referral CSTAR centers designed to prospectively follow patients with SLE-PAH diagnosed by right heart catheterization (RHC) [14, 15]. Using data from the CSTAR-PAH cohort, we aimed to develop a validated practical model for predicting long-term (i.e., 5-year) prognosis in individual patients with SLE-PAH.

## Materials and methods

The methods described in this article follow the Transparent Reporting of a multivariable prediction model for Individual Prognosis Or Diagnosis statement [16].

### Participants

We developed a prognostic model for all-cause death in the CSTAR-PAH cohort, which included patients with RHC-confirmed SLE-PAH from 14 referral centers participating in the CSTAR between November 2006 and May 2016 [15]. SLE was diagnosed by a rheumatologist at each CSTAR center in accordance with the 2012 Systemic Lupus International Collaborating Clinics classification criteria [17]. Diagnoses of PAH were based on RHC, defined as the mean pulmonary arterial pressure ≥ 25 mmHg at rest, pulmonary arterial wedge pressure ≤ 15 mmHg, and pulmonary vascular resistance (PVR) > 3 Wood units [5, 15]. Patients with other forms of pulmonary hypertension identified via a pulmonary function test showing total lung capacity < 60% and ventilation perfusion scintigraphy or computed tomographic pulmonary angiography showing pulmonary thromboembolism were excluded. We also excluded those with overlapping connective tissue diseases. The researchers at each center guaranteed the integrity and accuracy of their protocols and data, and approval from the medical ethics committee was obtained according to local regulations.

### Patient assessment and clinical outcomes

Baseline was defined as the time of SLE-PAH diagnosis confirmed by RHC. At baseline, we obtained information related to the following: demographic characteristics, medical history, physical examination findings, transthoracic echocardiography results, pulmonary function test results, hemodynamic measurements from RHC, and serum laboratory results. Planned and recorded comprehensive follow-up evaluations were arranged for patients every 3–12 months or earlier if there was a change in symptoms. Investigators, blinded to both variables and outcomes, reviewed and classified all clinical evaluations in a structured format. Data were collected independently from each participating center.

The endpoint of the present analysis was all-cause death during the follow-up period. The causes of death were ascertained by experienced rheumatologists at each referral center based on clinical records, social security data, and death registries. Deaths were assessed without knowledge of the candidate predictor variables.

### Candidate predictors

A multidisciplinary team of cardiologists, rheumatologists, and researchers selected the predictors for further evaluation in the prognostic model, based on existing literature and clinical judgment (see “Additional file 1: Appendix S1” for summary of candidate predictors) [18, 19]. From an initial list of 117 baseline predictor variables, 36 candidate predictors were selected (Additional file 1: Table S1) for further analyses. To improve model fit, all continuous predictors were first tested for normality and transformed appropriately if the association between a continuous predictor and the outcome was not linear [20] (see “Additional file 1: Appendix S2” for continuous variable transformation).

### Statistical analysis

Continuous variables are presented as medians with interquartile ranges, while categorical variables are presented as frequencies. The follow-up time was calculated from the date of baseline to the date of death from any cause or last follow-up before the end of the study period (February 1, 2020). For patients who were lost to follow-up, follow-up was censored at the date of last contact. Reverse Kaplan–Meier methods were used to estimate the median follow-up time [21]. All statistical analyses were performed using R statistical software version 3.4.3 (http://www.R-project.org/).

Missing data were explored to understand the patterns in value gaps, and those were considered to be missing at random. The values for the missing predictors were imputed using multiple imputation techniques based on chained equations (MICE) from R statistical software [22]. All candidate predictors with missing values were included in the multiple imputation model, together with the outcome, all related prespecified predictors (Additional file 1: Table S1) of the risk model, and the estimate of the cumulative hazard function [23]. A total of five imputed datasets were generated, and estimates obtained from the imputed datasets were combined using the Rubin rule [24].

To improve model accuracy and reduce model overfitting, we used the least absolute shrinkage and selection operator (LASSO) method [25] to select the most predictive variables from the preselected predictors. The optimal model was determined by cross-validation, sample size, and rationality of the predictors. Subsequently, the final model was developed using the Cox proportional hazards regression model [26]. The Cox proportional hazards assumption for each covariate was tested using Schoenfeld residuals [27] (e-Fig. 1). The 5-year all-cause death probability for an individual patient with SLE-PAH can be calculated using the following formula:

$${P}_{at\;5\,years}=1-{S}_{0 }{(t)}^{\text{exp}(\text{prognostic}\; \text{index})}$$

where S_0_(t) is the 5-year average survival probability, and the prognostic index equals the sum of the products of the predictors and their coefficients.

**Fig. 1:**
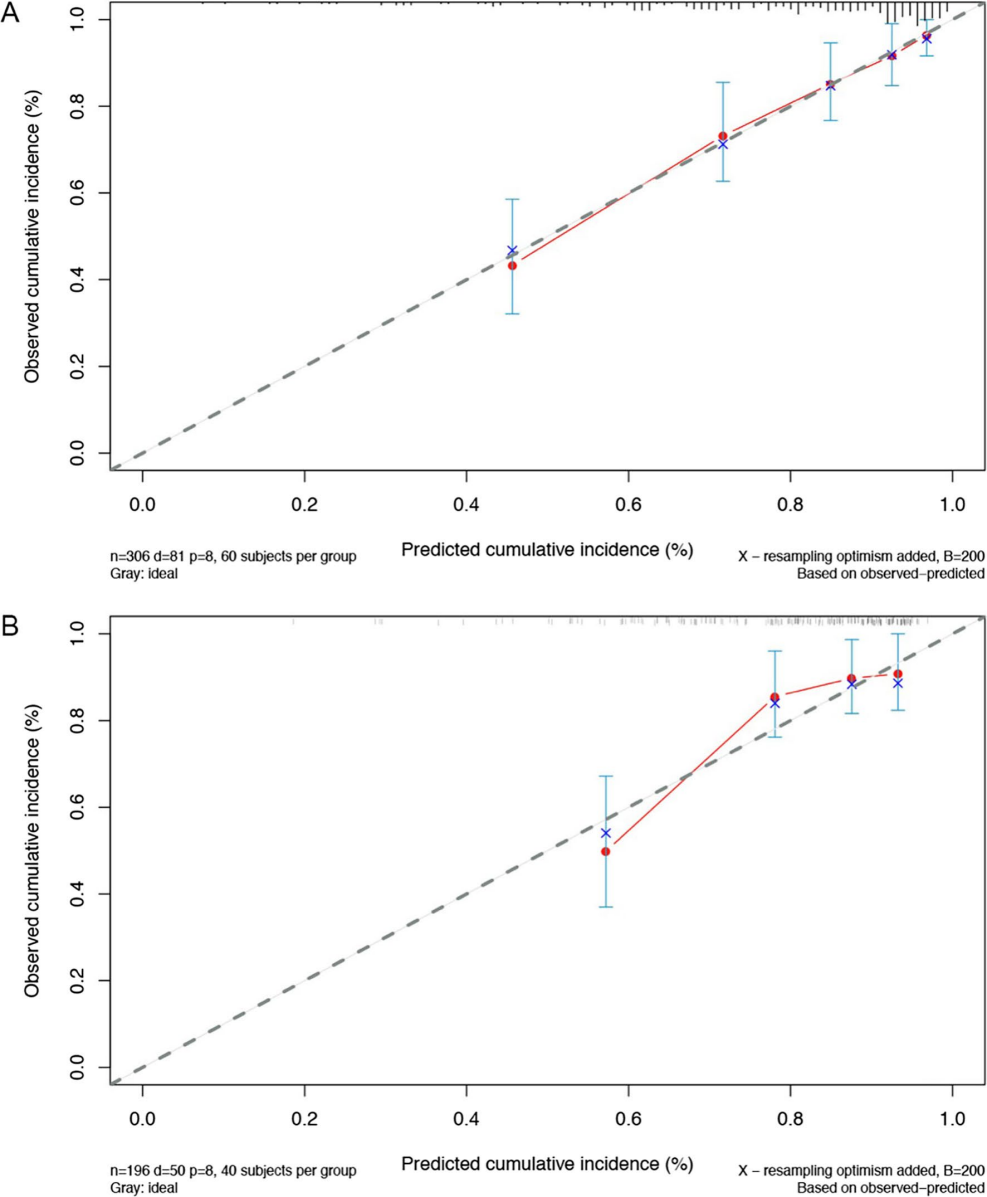
Calibration curve of the SLE-PAH prognostic model by comparing the observed vs predicted survival. **A** Imputed data; **B** complete data. SLE-PAH, systemic lupus erythematosus-associated pulmonary arterial hypertension

Bootstrapping was used to evaluate the performance of the model and permit adjustment for optimism. This is the most efficient internal validation procedure, as all aspects of model development, including variable selection, are validated [28]. For this purpose, 200 bootstraps were generated.

The predictive discrimination of this model was assessed using Harrell’s concordance index (C-index) [29]. Ensemble reliability was measured using the reliability component of the Brier score [30]. The observed and predicted hazards of all-cause death in patients with SLE-PAH were assessed using a calibration curve [31, 32]. In addition, patients were divided into five risk groups based on the cutoff points at the 20th, 40th, 60th, and 80th percentiles of probability distribution in the imputed dataset. Graphical comparisons of the observed and predicted all-cause deaths at 5 years according to the five risk groups were also performed. Finally, we developed easy-to-use measures to stratify risk. The final risk group was reassigned into high-, medium-, and low-risk groups based on the risk distributions of the five risk groups.

The REVEAL tools have powerful clinical applications in patients with PAH (World Health Organization [WHO] group 1(10). The discriminative power of the REVEAL prognostic Eq. (10) and simplified risk calculator [33] at 5 years were assessed (see “e-Appendix 3” for validation of the REVEAL model). The risk calculator’s ability to stratify risk at 5 years was also validated using Kaplan–Meier survival curves. Subsequently, we compared the performance of our SLE-PAH model with that of the REVEAL model.

## Results

### Clinical features and characteristics

The study cohort comprised 310 patients with RHC-confirmed SLE-PAH. Among them, 25.8% were newly diagnosed with PAH (The duration between onset of symptoms associated with PAH and the performance of RHC is within 3 months). The time from SLE-PAH onset to diagnosis by RHC was 1.5 years. The baseline clinical characteristics are shown in Table 1. Among the 310 patients, 306 with a confirmed mortality status were included in further analyses. During a median follow-up period of 1,615 patient-years, 81 deaths occurred. The median follow-up time using reverse Kaplan–Meier methods was 5.94 years (IQR 4.67–7.46), and 167 (54.6%) patients had ≥ 5 years of follow-up.

**Table 1:** Baseline clinical characteristics

Characteristic	Median (Q1-Q3) or no.(%)
Age at baseline	32.4 (27.9,41.0)
Female sex	304 (99.3%)
Raynaud phenomenon	155(50.7%)
Shortness of breath	257(84.0%)
Fatigue	94(30.7%)
Dry cough	60(19.6%)
Episodes of chest pain	40 (13.1%)
Syncope	25 (8.2%)
Palpitation	31 (10.1%)
Modified WHO functional class	-
I–II	146 (48.7%)
III–IV	151 (50.4%)
6MWD, m	425.0 (360.0, 480.0)
Pericardial effusion	144 (49.8%)
Mean right atrial pressure, mm Hg	5.0 (3.0, 8.0)
PVR, Wood units	9.3 (6.9, 14.2)
Cardiac index	2.7 (2.1,3.4)
Mean pulmonary artery pressure, mm Hg	46.0 (38.0, 54.0)
EGFR	108.1(90.3,120.9)
Acute/subacute cutaneous lupus	101 (33.0%)
Chronic cutaneous lupus	48 (15.7%)
Oral or nasal ulcers	63(20.6%)
Nonscarring alopecia	109(35.6%)
Arthritis	165(54.0%)
Serositis	108 (35.3%)
Renal disorder	103 (33.7%)
Neurologic disorder	12 (3.92%)
Hemolytic anemia	7(2.28%)
Thrombocytopenia	60 (19.7%)
Mild ILD	73 (24.8%)
Low complement	173 (56.5%)
Direct Coombs test	45 (14.7%)
Anti-Sm antibodies	105 (34.3%)
Anti-RNP antibodies	186 (61.6%)
NT-proBNP level	1074.0 (280.0,2254.0)
BNP level	186.0 (59.0,540.0)
NT-proBNP level/BNP	-
0 BNP < 50 /NT-proBNP < 300	207 (76.4%)
1 BNP > 50/ NT-proBNP > 300	64 (23.6%)
Dbil	3.5 (2.4, 5.0)
LDH	228.0 (186.0, 293.0)
Serum uric acid	342.0 (280.0, 456.0)
SLEDAI	4.0 (2.0, 9.0)

WHO, World Health Organization; 6MWD, 6-min walking distance; RHC, right heart catheterization; PVR, pulmonary vascular resistance; SLE, systemic lupus erythematosus; EGFR, estimated glomerular filtration rate; ILD, interstitial lung disease; NT-proBNP, N-terminal pro-brain natriuretic peptide; BNP, brain natriuretic peptide; Dbil, direct bilirubin level; LDH, lactate dehydrogenase; SLEDAI, systemic lupus erythematosus disease activity index

### Predictor variables

Eight variables with nonzero coefficients in the LASSO Cox regression model were selected to predict the risk of all-cause death, which included assessment of functional capacity, hemodynamic variables, organ involvement, and laboratory parameters. The definitions of the predictor variables are listed in Table 2. The coefficients of the eight variables in the five imputed datasets are listed in Additional file 1: Table S2.

**Table 2:** Definitions of the predictor variables in the final model

Predictor variable	Definition	Coding
Modified WHO functional class	Developed initially for heart failure by the New York Heart Association and adapted for PH by the WHO	Binary (Modified WHO functional class I/II 0, Modified WHO functional class III/IV 1)
6MWD	Six minute walk distance at baseline	Continuous (m)
PVR	It was determined at baseline based on the RHC as follows: PVR = ( mean pulmonary artery pressure − pulmonary artery wedge pressure) / cardiac output	Continuous (wood)
EGFR	It was assessed based on glomerular filtration rate, which was estimated using the Chronic Kidney Disease-Epidemiology Collaboration equation	Continuous (ml/minute/1.73 m^2^)
Thrombocytopenia	Thrombocytopenia (< 100,000/mm^3^) at least once in the absence of other known causes, such as drugs, portal hypertension, and thrombotic thrombocytopenic purpura	Binary (no 0, yes 1)
Mild ILD	Clinical diagnosis of ILD was based on the evidence of HRCT, other potential causes were excluded, such as infection, drug toxicity and heart failure. Mild ILD was defined as minimal disease on HRCT combined with total lung capacity > 60%	Binary (no 0, yes 1)
NT-proBNP level/BNP	NT-proBNP level or BNP level at baseline	Binary (BNP < 50 /NT-proBNP < 300 0, BNP > 50/ NT-proBNP > 300 1)
Dbil (log transformed)	Levels of serum direct bilirubin at baseline	Continuous (log transformed)

Abbreviations: WHO, World Health Organization; PH, Pulmonary arterial hypertension; 6MWD, 6-min walking distance; PVR, pulmonary vascular resistance; RHC, right heart catheterization; EGFR, estimated glomerular filtration rate; ILD, interstitial lung disease; HRCT, high-resolution computed tomography; N-terminal pro-brain natriuretic peptide; BNP, brain natriuretic peptide; Dbil, direct bilirubin level

### Model development and model performance

The entire follow-up data (306 patients with 81 events) were used to develop the prediction model. Hazard ratios and 95% confidence intervals were estimated by fitting the Cox proportional hazards model (Table 3). The cumulative risk of all-cause death in 5 years for an individual patient with SLE-PAH can be calculated using the following formula:

**Table 3:** Risk prediction model of SLE-PAH

Predictor variables	Risk prediction model			
	β coefficient	Hazard ratio	95% CI	*P* value
Modified WHO functional class	0.6106	1.8415	1.1134–3.0459	0.0174
6MWD	-0.0015	0.8586	0.6476–1.1383	0.2894
PVR	0.0538	1.4967	1.1125–2.0135	0.0077
EGFR	-0.0136	0.6641	0.4828–0.9135	0.0119
Thrombocytopenia	0.3233	1.3817	0.8251–2.3136	0.2190
Mild ILD	0.4907	1.6334	0.9930–2.6869	0.0533
NT-proBNP level/BNP	0.9482	2.5812	0.9379–7.1034	0.0664
Dbil (log transformed)	0.6751	1.6413	1.2521–2.1515	0.0003

Abbreviations: WHO, World Health Organization; 6MWD, 6-min walking distance; EGFR, estimated glomerular filtration rate; ILD, interstitial lung disease; NT-proBNP, N-terminal pro-brain natriuretic peptide; BNP, brain natriuretic peptide; Dbil, direct bilirubin level

$${P}_{at\;5\,years}=1-{0.9167271}^{\text{exp}(prognostic\;index)}$$

The prognostic index was calculated as follows: 0.6106 × modified WHO functional class – 0.0015 × 6MWD + 0.0528 × PVR – 0.0136 × EGFR + 0.3233 × thrombocytopenia + 0.4907 × mild ILD + 0.9482 × NT-proBNP/BNP + 0.6751 × Dbil (log transformed), where 6MWD is six-minute walking distance, EGFR is estimated glomerular filtration rate, ILD is interstitial lung disease, NT-proBNP/BNP is N-terminal pro-brain natriuretic peptide / brain natriuretic peptide level, and Dbil is direct bilirubin level. The coding of the predictors is presented in Table 2.

The model performance of a 5-year predicted risk was assessed using 306 patients with 65 events. The apparent C-index of the model was 0.78. After enhanced bootstrap adjustment for optimism, the prediction model had an optimism-corrected C-index of 0.77 and an optimism-corrected Brier score of 0.04 (Table 4). A calibration plot with 200 bootstrap replications showed a comparison between the predicted and observed risks (Fig. 1). Sensitivity analyses with complete data were also performed (see “Additional file 1: Appendix S4” and Additional file 1: Table S3 for sensitivity analyses).

**Table 4:** Model performance

Performance measure	Apparent cohort performance	Average optimism calculated from 200 bootstrap validation	Optimism-corrected performance
Total C-index	0.79	0.02	0.77
C-index at 5 years	0.80	0.03	0.77
Brier score	0.13	0.01	0.14

e-Fig. 2 illustrates a good agreement between the observed and predicted risk of all-cause death at 5 years in the stacked imputed dataset and the complete original dataset. According to the average risk probabilities of the five groups and consideration of clinical application, groups 2–4 were merged into one group. Finally, the predicted risk was reassigned as low risk (< 0.05), medium risk (0.05–0.38), and high risk (> 0.38). The clinical implications of the model were verified in both the stacked imputed dataset and the complete original dataset (Fig. 2).

**Fig. 2:**
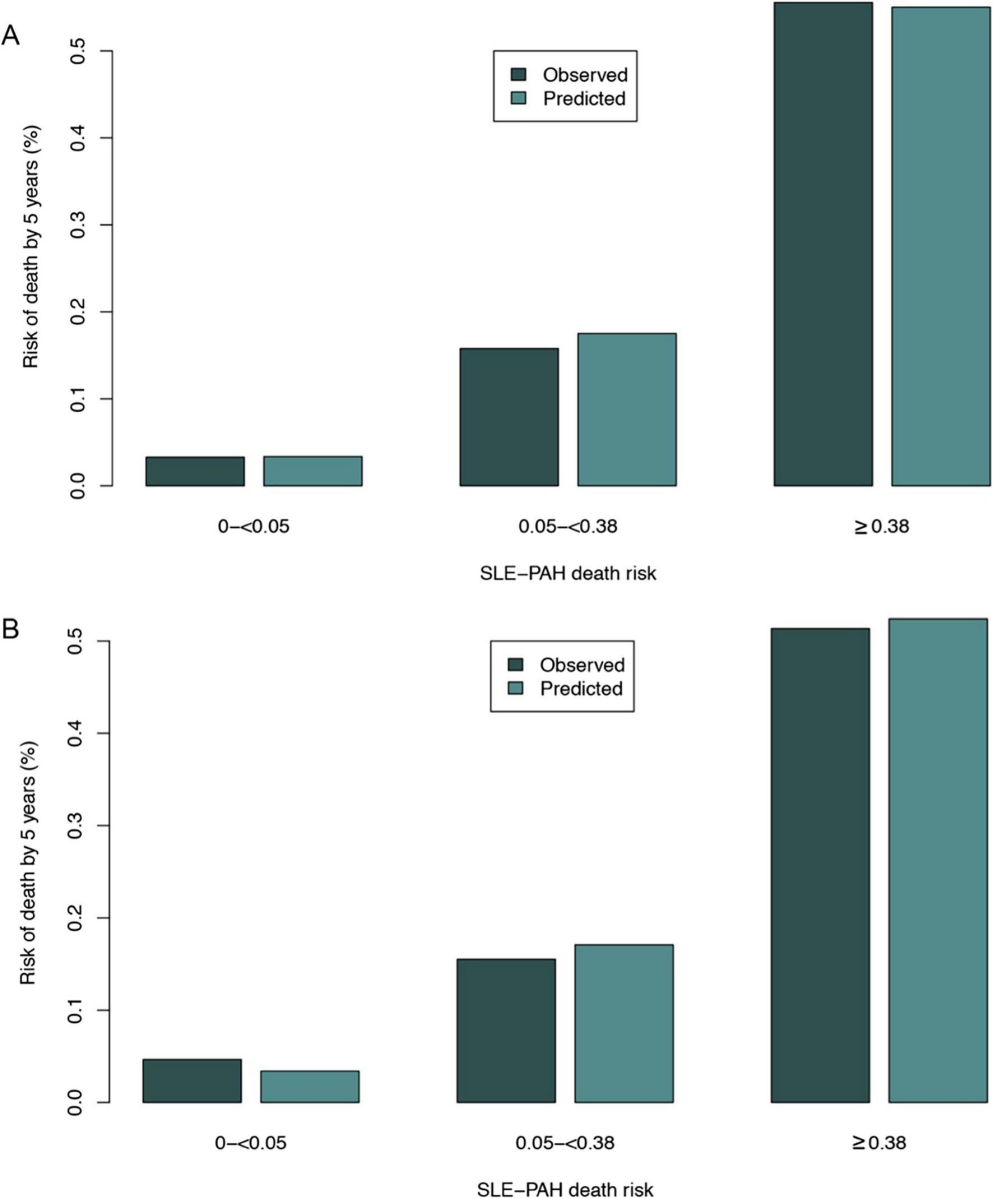
Comparison of observed and predicted risks according to three risk groups of the SLE-PAH prognostic model. Vertical bars represent observed and model-based predicted probability of all-cause death by 5 years. **A** Imputed data; **B** complete data. SLE-PAH, systemic lupus erythematosus-associated pulmonary arterial hypertension

### Comparison with the REVEAL prognostic model

**Fig. 3:**
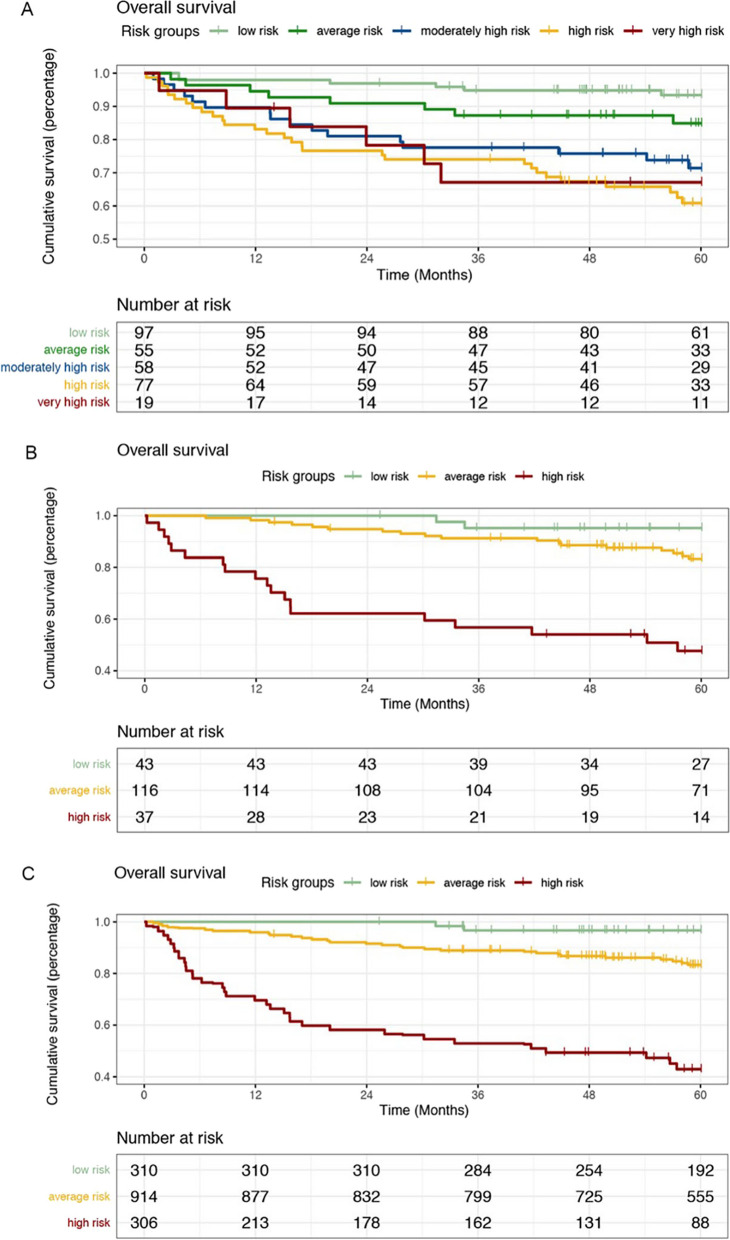
Survival by risk group according to the **A** REVEAL simplified risk calculator, **B** our purposed SLE-PAH prognostic model using complete data and **C** our purposed SLE-PAH prognostic model using imputed data. REVEAL, Registry to Evaluate Early and Long-Term PAH Disease Management; SLE-PAH, systemic lupus erythematosus-associated pulmonary arterial hypertension

The C-index for the prognostic equation and risk score calculator in the SLE-PAH cohort was 0.71 and 0.70, respectively, indicating poorer but modest discriminatory ability compared with that of the SLE-PAH prediction model. However, the Kaplan–Meier survival curves of the five groups classified by the REVEAL risk score calculator showed that the risk groups did not predict risk accurately, especially in the very high risk group (Fig. 3). The REVEAL model seemed to be more appropriate for discriminating low risk, average risk, and the rest (including moderately high risk, high risk, and very high risk). According to the Kaplan–Meier survival curves classified by the SLE-PAH prediction model, there was a significant difference among the three risk groups.

## Discussion

To our knowledge, this study provides the first prognostic model for all-cause death in patients with SLE-PAH, based on the largest, prospectively recruited, national SLE-PAH cohort. The model was based on eight objectively measured and readily available variables: modified WHO functional class, 6MWD, PVR, EGFR, thrombocytopenia, mild ILD, NT-proBNP/BNP, and Dbil. Three risk groups were identified based on predicted probability and clinical applicability. The internal validation of the model showed its robustness and adequate performance.

Our study had several advantages. First, to provide a comprehensive evaluation of SLE-PAH, the candidate predictors were selected not only based on the known prognostic factors for PAH but also by considering the clinical features and assessment of SLE. The candidate predictors were confirmed by expert opinion to ensure objectivity and stability [16]. Subsequently, the LASSO method was used to reduce the dimensions and statistically control for potential selection bias. It surpasses the method of choosing predictors based on the strength of their univariable association with the outcome. Second, the model was built from easily accessible variables with a specific formula, which means it can be straightforwardly applied in clinical settings and is readily amenable to external validation. Third, we may keep the variables as continuous if they show a linear relationship with the outcome, because categorization of predictors causes a loss in information [20], which will lead to existing algorithms having a low positive predictive accuracy for outcome.

The new SLE-PAH model we have developed showed good discrimination with satisfactory calibration between the expected and observed risks, whereas the REVEAL model might not particularly suitable for patients with SLE-PAH [5]. REVEAL tools have been extensively validated and have performed well in geographically diverse PAH populations [33–35]. However, as one of the etiologies of PAH, SLE is an autoimmune disease characterized by lesions in multiple organs [12].To effectively predict the death of SLE-PAH, both multi-organ involvement and PAH-related right ventricular failure need to be considered. Besides, patients with SLE-PAH were younger, overwhelmingly female. Sex differences can lead to inaccuracies when we use REVEAL to predict the prognosis of SLE-PAH [36]. Therefore, limitations may remain when assessing SLE-PAH. This study showed that when applied to SLE-PAH, the REVEAL prognostic model had limited discriminatory power (C-index statistics, 0.71 and 0.70), which may lead to inaccurate stratification of risk groups, especially the moderately high risk, high-risk, and very high risk groups. Additional recommendations for the risk assessment of PAH include the 2015/2022 ESC/ERS guidelines on PAH [5, 6] and Results From the Comparative, Prospective Registry of Newly Initiated Therapies for Pulmonary Hypertension (COMPERA) [37]. Nevertheless, these recommendations are primarily expert consensus-based risk stratification tools rather than rigorously derived models. Consequently, we encountered limitations in our ability to calculate the risk probability and make comparisons with our own model.

Previous studies have assessed the performance of various predictors of PAH prognosis. The clinical plausibility, feasibility, and applicability of the final selected predictors were further confirmed by expert opinion. The modified WHO functional class, 6MWD, NT-proBNP/BNP, Dbil, and PVR are well-recognized predictors and have been confirmed to be associated with PAH prognosis [5, 10, 38–45]. Moreover, previous studies have shown that PVR might be a measure that is sensitive to treatment effects [41]. In this study, we confirmed that PVR was associated with a continuous increase in the risk of death due to SLE-PAH, and its potential clinical application is worth further exploration. Notably, our model development approach took into account the multisystem clinical manifestations in patients with SLE, including EGFR, thrombocytopenia, and mild ILD. Multisystem involvement may contribute to the development of more severe forms of the disease and yield a poor prognosis. Nevertheless, prognostic information from systematic evaluation of patients with SLE-PAH more closely mirrors actual practice.

Risk stratification is helpful in identifying patients who may benefit from intensive therapy, which would improve clinical decision-making. Both complete case analysis and imputed data analysis showed that the mortality rate of SLE-PAH was > 50% in the high-risk group, and some of the patients were censored during the follow-up. Given the good response to immunosuppressive therapy in patients with SLE-PAH [2, 46], clinical strategies to reinforce b both immunosuppressive and PAH target therapy may be particularly beneficial in high-risk patients. Of course, the intention of the proposed SLE-PAH prognosis model is not to replace physicians’ clinical judgment but rather to complement clinical reasoning by providing objective individualized prognostic information. Specific clinical decision-making needs further investigation. The ability to prospectively identify high-risk patients represents the first step in improving outcomes. However, we still need to treat prediction probability as a continuum, interpreted within each patient’s clinical context, since the model does not categorize patients into simple high- or low-risk groups with predefined therapeutic strategies (Additional file 2).

This study has several limitations. The cohorts consisted of patients with SLE-PAH, recruited on the basis of prespecified criteria, which may limit the use of the model in all patients with SLE-PAH. We did not include patients who had only been examined using transthoracic echocardiography instead of RHC. However, according to the ESC/ERS recommendation, the diagnosis of PAH should be confirmed by definitive RHC [5]. In addition, most of the baseline data were collected at an early stage in the course of SLE-PAH. The model was better used in patients with disease stages similar to those of the study cohort. The applicability to reassessment of risk at any point in the course of this model needs further validation.

Because PAH is a relatively rare complication of SLE and the mortality rates have declined significantly in recent years, all samples in the CSTAR-PAH cohort were used to develop a specific SLE-PAH model. We have not yet obtained a separate dataset of patients with SLE-PAH for external validation. The generalizability of the results, especially to other regions and races, should be carefully considered. Further research is required to confirm our proposed models and to measure their performance.

This study presents a retrospective analysis of the prospective CSTAR PAH cohort. It is important to note that a limited amount of data is not derived from standardized prospective clinical evaluations. Consequently, we have incorporated a portion of missing data, which can be attributed to challenges in acquiring certain investigations and inconsistencies in specific reports across various centers. We addressed this problem by using a multiple imputation method, which is widely regarded as the best approach. However, the mechanisms underlying the missing pattern may be complicated and may bias the estimates. A study with more complete data is needed in the future to obtain accurate estimates.

## Conclusion

The risk prediction model proposed in this study provides individualized estimates of risk regarding all-cause death in SLE-PAH, which should be used by physicians experienced in the management of the condition. By obtaining an evidence-based assessment of the patient, clinicians may be better able to individualize and optimize therapeutic strategies to improve survival. External validation will be required to demonstrate the accuracy of this model in different cohorts of SLE-PAH.

### Supplementary Information

**Additional file 1:** **Appendix S1.** (Summary of candidate predictors), **Appendix S2.** (Continuous variable transformation), **Appendix S3. **(Validation of the REVEAL model), **Appendix S4.** (Sensitivity analyses), **Fig.S1. **(The Cox proportional hazards assumption for each covariate), **Fig. S2.** (Comparison of observed and predicted risk by 5 risk groups of the SLE-PAH prognostic model), **Table S1.** (Predictors for multiple imputation model and LASSO regression model), **Table S2.** (Least absolute shrinkage and selection operator (LASSO) coefficient of the five imputed datasets), **Table S3.** Sensitivity analysis of SLE-PAH prediction model using complete cases only**.** Supplementary Information of this study.

**Additional file 2. **TRIPOD checklist. TRIPOD checklist: Prediction Model Development and Validation**.** TRIPOD checklist of this study.

## Data Availability

The datasets analyzed during the current study are not publicly available due to the data also forms part of an ongoing study, but are available from the corresponding author on reasonable request.

## Abbreviations

BNP: Brain natriuretic peptide
CSTAR: The Chinese SLE Treatment and Research Group
Dbil: Direct bilirubin level
EGFR: Estimated glomerular filtration rate
ILD: Interstitial lung disease
LASSO: Least absolute shrinkage and selection operator
NT-proBNP: N-terminal pro-brain natriuretic peptide
PAH: Pulmonary arterial hypertension
PVR: Pulmonary vascular resistance
REVEAL: Registry to Evaluate Early and Long-Term PAH Disease Management
RHC: Right heart catheterization
SLE: Systemic lupus erythematosus
6MWD: Six-minute walking distance
