# Supplementary material for: A prognostic model for systemic lupus erythematosus-associated pulmonary arterial hypertension: CSTAR-PAH cohort study
Source: Respir Res. 2023 Sep 9;24:220. doi: 10.1186/s12931-023-02522-2 (PMC10492375; doi:10.1186/s12931-023-02522-2)
Supplement: Supplementary file 1 — Additional file 1: Appendix S1. (Summary of candidate predictors), Appendix S2. (Continuous variable transformation), Appendix S3. (Validation of the REVEAL model), Appendix S4. (Sensitivity analyses), Fig.S1. (The Cox proportional hazards assumption for each covariate), Fig. S2. (Comparison of observed and predicted risk by 5 risk groups of the SLE-PAH prognostic model), Table S1. (Predictors for multiple imputation model and LASSO regression model), Table S2. (Least absolute shrinkage and selection operator (LASSO) coefficient of the five imputed datasets), Table S3. Sensitivity analysis of SLE-PAH prediction model using complete cases only. Supplementary Information of this study. [file 12931_2023_2522_MOESM1_ESM.docx]

Supplemental Material

A prognostic model for systemic lupus erythematosus-associated pulmonary arterial hypertension: A CSTAR-PAH cohort study

**e-Appendix**

e-Appendix 1

Summary of candidate predictors

A review of the risk assessment tools and prognostic scores in managing patients with pulmonary arterial hypertension (PAH) was performed in 2019 to identify risk factors with sufficient evidence for inclusion as predictor variables in the risk model (1). The review was based on expert consensus regarding PAH. A meta-analysis confirmed that some of these predictors are survival and prognostic factors for systemic lupus erythematosus-associated pulmonary arterial hypertension (SLE-PAH) (2). As SLE-PAH is a unique and distinct disease subtype, the predictor from the prognostic study of SLE-PAH was included (3). We also added predictors not included in prior literature but related to SLE disease severity, activity, or SLE-PAH that we were able to measure reliably in our data, forming the prediction model’s candidate variables of interest, which are intended to be used to predict all-cause death in patients with SLE-PAH.

e-Appendix 2

Continuous variable transformation

Data distribution was assessed using histograms. The possible nonlinear relationship between each continuous variable and death was evaluated using restricted cubic spline regression. The distributions of pro-brain natriuretic peptide (BNP), N-terminal pro-brain natriuretic peptide (NT-proBNP), total bilirubin (Tbil), direct bilirubin (Dbil), cardiac output (CO), mean pulmonary artery pressure (mPAP), age, and systemic lupus erythematosus disease activity index (SLEDAI) were skewed and had a nonlinear relationship with mortality. Therefore, these parameters were log-transformed to normalize their distribution. After logarithmic transformation, BNP, NT-proBNP, Tbil, Dbil, CO, and mPAP showed a linear relationship with mortality, while age and SLEDAI did not. To ensure that the model meets the assumptions of linear regression, age and SLEDAI were converted into categorical predictors according to curvilinear relationships.

e-Appendix 3

Validation of the REVEAL model

The Registry to Evaluate Early and Long-Term PAH Disease Management (REVEAL) prognostic equation and simplified risk calculator were designed to predict 1-year survival in patients with PAH. However, our study focused on the long-term survival of patients with SLE-PAH. Therefore, the predictive value of REVEAL tools using 5-year outcomes was validated in this study. Since S_0_(1) indicates the baseline survival at 1 year, the individual’s predicted probability of 5-year survival cannot be calculated. The calibration of the prognostic equation (4) and risk stratification of the prognostic equation cannot be validated.

The REVEAL prognostic equation and simplified risk calculator were assessed in the Chinese SLE Treatment and Research Group (CSTAR)-PAH cohort in accordance with previously described methods (5, 6). The follow-up of patients was censored 5 years after baseline. The discriminative and predictive abilities of the prognostic equation and simplified risk calculator were assessed based on Harrell’s concordance index (C-index) (7). We also validated the risk calculator’s ability for risk stratification. Kaplan-Meier survival curves were constructed to analyze survival differences among the five risk groups.

e-Appendix 4

Sensitivity analyses

We conducted sensitivity analyses using the complete data and the imputed data to examine the robustness of the regression models. Because no major differences in the magnitude or direction of the effect estimates were observed between the analyses using imputed data and complete cases only (n=196) (e-Table 3), we simply presented the results based on the imputed datasets.

**e-Figure**

e-Figure 1 The Cox proportional hazards assumption for each covariate.

WHO, modified WHO functional class; 6MWT, 6-minute walking distance test; PVR, pulmonary vascular resistance; EGFR, estimated glomerular filtration rate; ILD, interstitial lung disease; NT-proBNP/BNP, N-terminal pro-brain natriuretic peptide / brain natriuretic peptide level; logDbil, log-transformed direct bilirubin level.

**
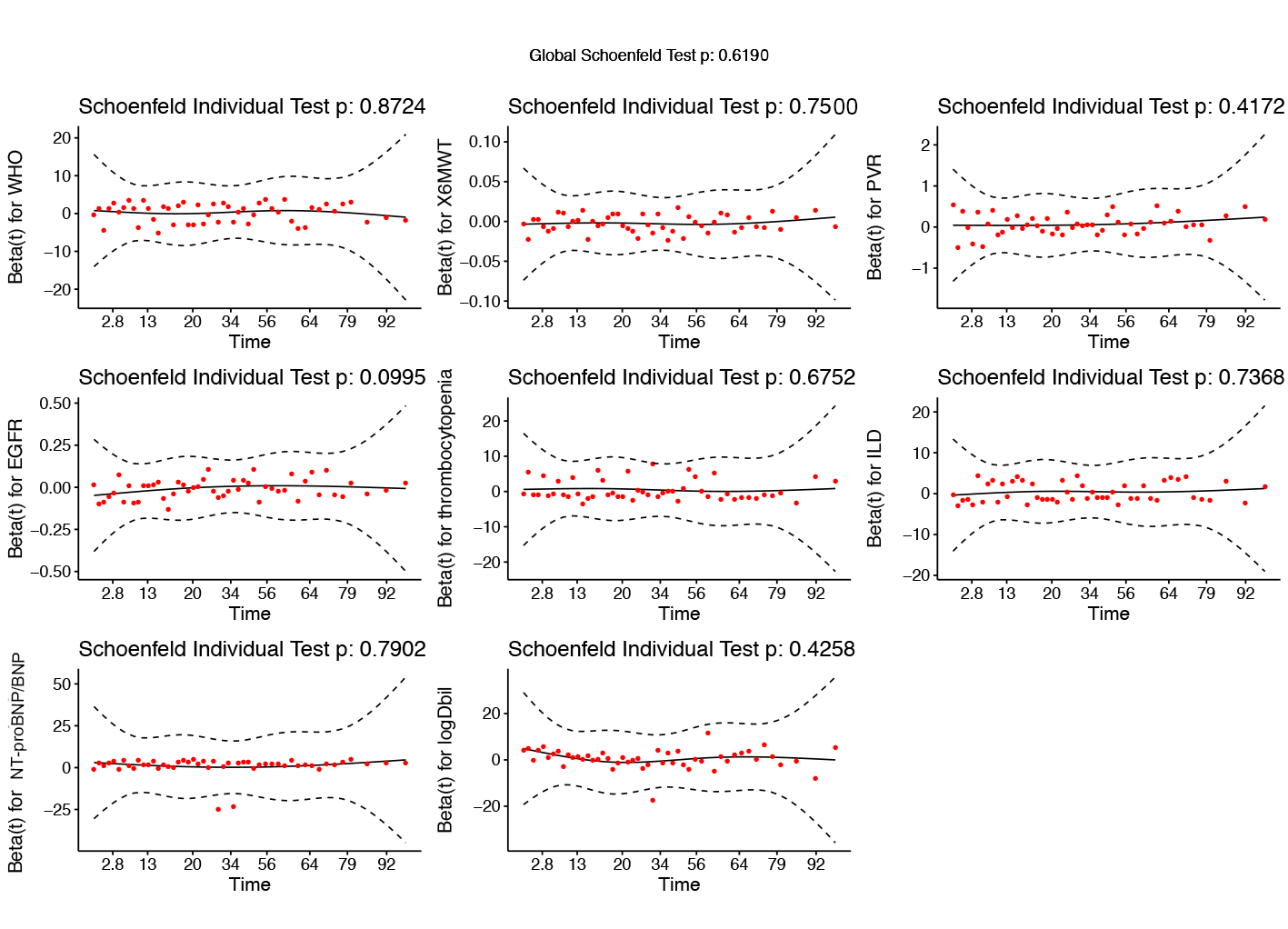
**

e-Figure 2 Comparison of observed and predicted risk by 5 risk groups of the SLE-PAH prognostic model.

**
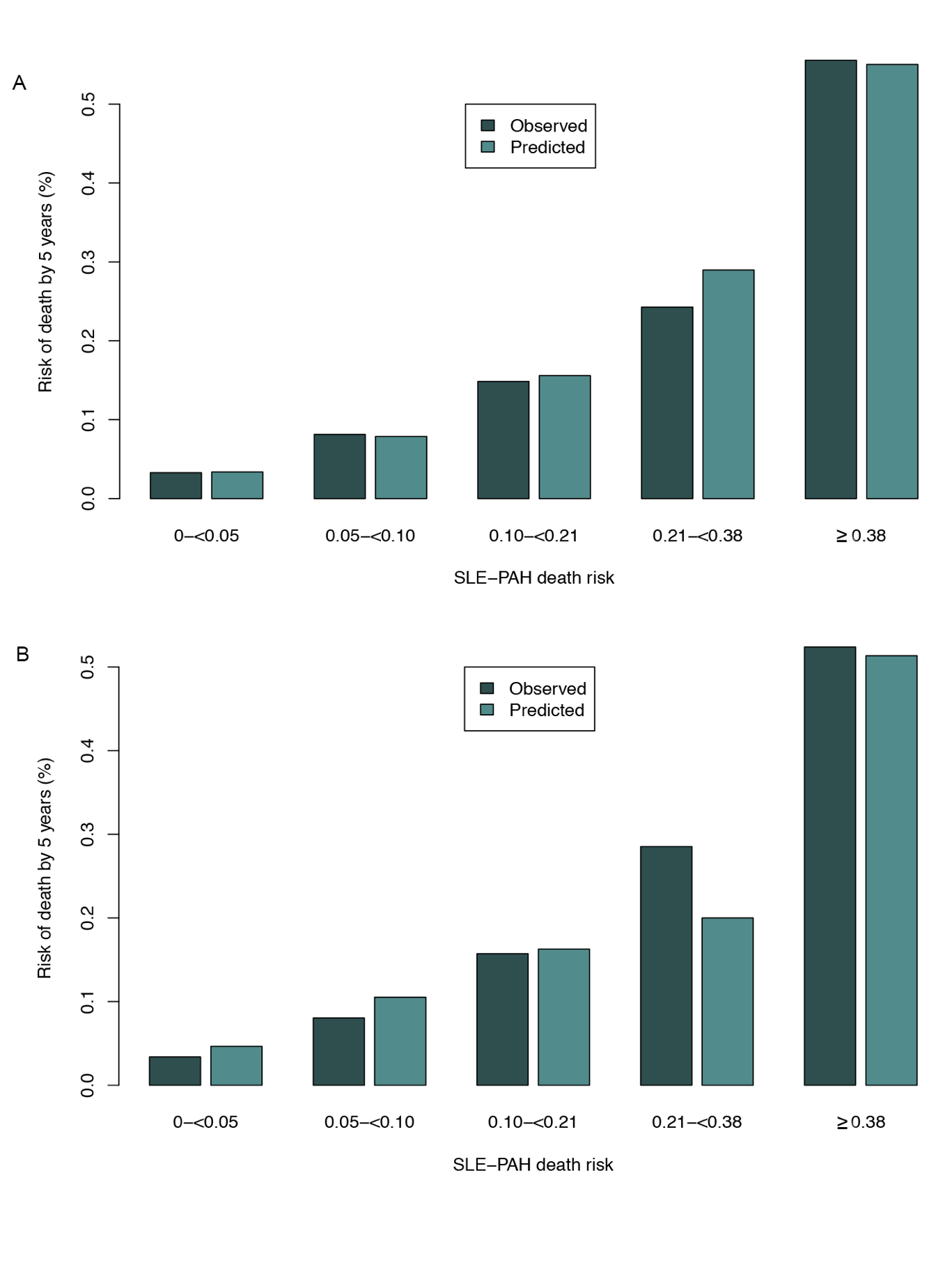
**(A) imputed data (B) complete data.

**e-Table**

e-Table 1. Predictors for multiple imputation model and LASSO regression model

| Category | Prespecified predictors for multiple imputation | Candidate predictors for  LASSO regression model |
| --- | --- | --- |
| Demographics | Age, sex | Age (categorical), sex |
| Symptoms | Raynaud phenomenon, shortness of breath, fatigue, dry cough, episodes of chest pain, syncope, palpitation | Raynaud phenomenon, shortness of breath, fatigue, dry cough, episodes of chest pain ,syncope, palpitation |
| Assessment of Functional Capacity | Modified WHO functional class, 6MWD, | Modified WHO functional class, 6MWD, |
| Transthoracic Tchocardiography | pericardial effusion, PASP | pericardial effusion |
| Hemodynamic Variables (RHC) | Mean right atrial pressure, pulmonary vascular resistance, cardiac index, cardiac output, mean pulmonary artery pressure | Mean right atrial pressure, mean pulmonary artery pressure, cardiac index, mean pulmonary artery pressure(log) |
| SLE related Clinical features | EGFR, acute/subacute cutaneous lupus, chronic cutaneous lupus, oral or nasal ulcers, nonscarring alopecia, arthritis, serositis, renal disorder, neurologic disorder, hemolytic anemia, thrombocytopenia, mild ILD, low complement, C3, C4 | EGFR, acute/subacute cutaneous lupus, chronic cutaneous lupus, oral or nasal ulcers, nonscarring alopecia, arthritis, serositis, renal disorder, neurologic disorder, hemolytic anemia, thrombocytopenia, mild ILD, low complement |
| Auto-antibodies | Direct Coombs test, anti-Sm antibodies, anti-RNP antibodies | Direct Coombs test , anti-Sm antibodies, anti-RNP antibodies |
| Lab Values | NT-proBNP level, BNP level, NT-proBNP level or BNP level, Dbil, Tbil, LDH, UA | NT-proBNP level or BNP level, UA, Dbil(log) |
| Disease activity | SLEDAI | SLEDAI(categorical) |

Abbreviations: WHO, World Health Organization; 6MW, 6-minute walking distance; PASP, pulmonary artery systolic pressure; RHC, right heart catheterization; EGFR, estimated glomerular filtration rate; mild ILD, interstitial lung disease; C3, complement component 3; C4, complement component 4; NT-proBNP, N-terminal pro-brain natriuretic peptide; BNP, brain natriuretic peptide; Dbil, direct bilirubin level; Tbil, total bilirubin level; LDH, lactate dehydrogenase; UA, serum uric acid

e-Table 2. Least absolute shrinkage and selection operator (LASSO) coefficient of the five imputed datasets

|  | coefficient | | | | |
| --- | --- | --- | --- | --- | --- |
|  | The first imputed dataset | The second imputed dataset | The third imputed dataset | The forth imputed dataset | The fifth imputed dataset |
| Modified WHO functional class* | 0.246214 | 0.361475 | 0.261611 | 0.268678 | 0.311003 |
| 6MWD* | -0.001659 | -0.000051 | -0.001974 | -0.001541 | -0.000614 |
| Mean right atrial pressure | 0.011947 | 0 | 0 | 0 | 0 |
| Pulmonary vascular resistance* | 0.044171 | 0.064446 | 0.031177 | 0.051574 | 0.051023 |
| Cardiac index | 0 | 0 | -0.048666 | 0 | -0.011962 |
| EGFR* | -0.007158 | -0.009404 | -0.007279 | -0.007325 | -0.008142 |
| Nonscarring alopecia | -0.006336 | 0 | -0.016790 | 0 | 0 |
| Thrombocytopenia* | 0.053460 | 0.046150 | 0.134754 | 0.071917 | 0.045636 |
| Mild ILD* | 0.302271 | 0.276682 | 0.262986 | 0.264199 | 0.231374 |
| NT-proBNP level or BNP level* | 0.275474 | 0.043129 | 0.342967 | 0.257490 | 0.245334 |
| Mean pulmonary artery pressure (log) | 0.243828 | 0 | 0.799282 | 0.274059 | 0.291699 |
| Dbil (log)* | 1.264023 | 1.258835 | 1.221815 | 1.183828 | 1.263196 |

Abbreviations: WHO, World Health Organization; 6MWD, 6-minute walking distance; EGFR, estimated glomerular filtration rate; ILD, interstitial lung disease; NT-proBNP, N-terminal pro-brain natriuretic peptide; BNP, brain natriuretic peptide; Dbil, direct bilirubin level

*The overlapped predictors with coefficients in all five imputed datasets were considered as the predictors for the final model.

e-Table 3. Sensitivity analysis of SLE-PAH prediction model using complete cases only

| Predictor variables | Risk prediction model | | | |
| --- | --- | --- | --- | --- |
|  | β coefficient | Hazard Ratio | 95%CI | *P* value |
| Modified WHO functional class | 0.304964 | 1.3566 | 0.7035 - 2.616 | 0.3627 |
| 6MWD | -0.002173 | 0.9978 | 0.9947 - 1.001 | 0.1721 |
| Pulmonary vascular resistance | 0.075083 | 1.0780 | 1.0148 - 1.145 | 0.0149 * |
| EGFR | -0.007022 | 0.9930 | 0.9785 - 1.008 | 0.3506 |
| Thrombocytopenia | 0.491999 | 1.6356 | 0.8088 - 3.307 | 0.1709 |
| Mild ILD | 0.402742 | 1.4959 | 0.8146 - 2.747 | 0.1940 |
| NT-proBNP level/BNP | 1.289863 | 3.6323 | 0.8241 - 16.009 | 0.0883 |
| Dbil (log transformed) | 0.687265 | 1.9883 | 0.6806 - 5.809 | 0.2089 |
| Optimism-corrected total C-index | 0.75 | | | |
| Optimism-corrected C-index at 5 years | 0.75 | | | |
| Optimism-corrected Brier score | 0.14 | | | |

Abbreviations: WHO, World Health Organization; 6MWD, 6-minute walking distance; EGFR, estimated glomerular filtration rate; ILD, interstitial lung disease; NT-proBNP, N-terminal pro-brain natriuretic peptide; BNP, brain natriuretic peptide; Dbil, direct bilirubin level

**References**

1. Kanwar M, Raina A, Lohmueller L, Kraisangka J, Benza R: The Use of Risk Assessment Tools and Prognostic Scores in Managing Patients with Pulmonary Arterial Hypertension. Curr Hypertens Rep 2019;21:45.

2. Qian J, Wang Y, Huang C, et al.: Survival and prognostic factors of systemic lupus erythematosus-associated pulmonary arterial hypertension: A PRISMA-compliant systematic review and meta-analysis. Autoimmunity Reviews 2016;15:250-7.

3. Hachulla E, Jais X, Cinquetti G, et al.: Pulmonary Arterial Hypertension Associated With Systemic Lupus Erythematosus: Results From the French Pulmonary Hypertension Registry. Chest 2018;153:143-51.

4. Van Calster B, McLernon DJ, van Smeden M, et al.: Calibration: the Achilles heel of predictive analytics. BMC Med 2019;17:230.

5. Benza RL, Miller DP, Gomberg-Maitland M, et al.: Predicting Survival in Pulmonary Arterial Hypertension. Circulation 2010;122:164-72.

6. Benza RL, Gomberg-Maitland M, Miller DP, et al.: The REVEAL Registry risk score calculator in patients newly diagnosed with pulmonary arterial hypertension. Chest 2012;141:354-62.

7. FE H, KL L, DB M: Multivariable prognostic models: issues in developing models, evaluating assumptions and adequacy, and measuring and reducing errors. Statistics in medicine 1996;15:361-87.

| **Section/Topic** | **Item** |  | **Checklist Item** | **Page** |
| --- | --- | --- | --- | --- |
| **Title and abstract** | | | | |
| Title | 1 | D;V | Identify the study as developing and/or validating a multivariable prediction model, the target population, and the outcome to be predicted. | 1 |
| Abstract | 2 | D;V | Provide a summary of objectives, study design, setting, participants, sample size, predictors, outcome, statistical analysis, results, and conclusions. | 4 |
| **Introduction** | | | | |
| Background and objectives | 3a | D;V | Explain the medical context (including whether diagnostic or prognostic) and rationale for developing or validating the multivariable prediction model, including references to existing models. | 7 |
|  | 3b | D;V | Specify the objectives, including whether the study describes the development or validation of the model or both. | 7 |
| **Methods** | | | | |
| Source of data | 4a | D;V | Describe the study design or source of data (e.g., randomized trial, cohort, or registry data), separately for the development and validation data sets, if applicable. | 8 |
|  | 4b | D;V | Specify the key study dates, including start of accrual; end of accrual; and, if applicable, end of follow-up. | 8、9 |
| Participants | 5a | D;V | Specify key elements of the study setting (e.g., primary care, secondary care, general population) including number and location of centres. | 8 |
|  | 5b | D;V | Describe eligibility criteria for participants. | 8 |
|  | 5c | D;V | Give details of treatments received, if relevant. | N/A |
| Outcome | 6a | D;V | Clearly define the outcome that is predicted by the prediction model, including how and when assessed. | 9 |
|  | 6b | D;V | Report any actions to blind assessment of the outcome to be predicted. | 9 |
| Predictors | 7a | D;V | Clearly define all predictors used in developing the multivariable prediction model, including how and when they were measured. | 9、e-Appendix |
|  | 7b | D;V | Report any actions to blind assessment of predictors for the outcome and other predictors. | 8 |
| Sample size | 8 | D;V | Explain how the study size was arrived at. | N/A |
| Missing data | 9 | D;V | Describe how missing data were handled (e.g., complete-case analysis, single imputation, multiple imputation) with details of any imputation method. | 10 |
| Statistical analysis methods | 10a | D | Describe how predictors were handled in the analyses. | 10、11、e-Appendix |
|  | 10b | D | Specify type of model, all model-building procedures (including any predictor selection), and method for internal validation. | 9、10、e-Appendix |
|  | 10c | V | For validation, describe how the predictions were calculated. | N/A |
|  | 10d | D;V | Specify all measures used to assess model performance and, if relevant, to compare multiple models. | 11 |
|  | 10e | V | Describe any model updating (e.g., recalibration) arising from the validation, if done. | N/A |
| Risk groups | 11 | D;V | Provide details on how risk groups were created, if done. | 11 |
| Development vs. validation | 12 | V | For validation, identify any differences from the development data in setting, eligibility criteria, outcome, and predictors. | N/A |
| **Results** | | | | |
| Participants | 13a | D;V | Describe the flow of participants through the study, including the number of participants with and without the outcome and, if applicable, a summary of the follow-up time. A diagram may be helpful. | 12 |
|  | 13b | D;V | Describe the characteristics of the participants (basic demographics, clinical features, available predictors), including the number of participants with missing data for predictors and outcome. | 12、13 |
|  | 13c | V | For validation, show a comparison with the development data of the distribution of important variables (demographics, predictors and outcome). | N/A |
| Model development | 14a | D | Specify the number of participants and outcome events in each analysis. | 12 |
|  | 14b | D | If done, report the unadjusted association between each candidate predictor and outcome. | N/A |
| Model specification | 15a | D | Present the full prediction model to allow predictions for individuals (i.e., all regression coefficients, and model intercept or baseline survival at a given time point). | 12 |
|  | 15b | D | Explain how to use the prediction model. | 12、13 |
| Model performance | 16 | D;V | Report performance measures (with CIs) for the prediction model. | 13、28 |
| Model-updating | 17 | V | If done, report the results from any model updating (i.e., model specification, model performance). | N/A |
| **Discussion** | | | | |
| Limitations | 18 | D;V | Discuss any limitations of the study (such as nonrepresentative sample, few events per predictor, missing data). | 16、17 |
| Interpretation | 19a | V | For validation, discuss the results with reference to performance in the development data, and any other validation data. | N/A |
|  | 19b | D;V | Give an overall interpretation of the results, considering objectives, limitations, results from similar studies, and other relevant evidence. | 15-17 |
| Implications | 20 | D;V | Discuss the potential clinical use of the model and implications for future research. | 16 |
| **Other information** | | | | |
| Supplementary information | 21 | D;V | Provide information about the availability of supplementary resources, such as study protocol, Web calculator, and data sets. | e-Appendix |
| Funding | 22 | D;V | Give the source of funding and the role of the funders for the present study. | 3 |
